# Polymorphisms of Leptin-b Gene Associated with Growth Traits in Orange-Spotted Grouper (*Epinephelus coioides*)

**DOI:** 10.3390/ijms150711996

**Published:** 2014-07-07

**Authors:** Hai Huang, Yun Wei, Zining Meng, Yong Zhang, Xiaochun Liu, Liang Guo, Jian Luo, Guohua Chen, Haoran Lin

**Affiliations:** 1 Sanya Science & Technology Academy of Hainan National Breeding and Multiplication, Sanya 572000, China; E-Mail: huanghai74@126.com; 2 Key Laboratory of Tropical Biology Resources, Ministry of Education, College of Ocean, Hainan University, Haikou 570228, China; E-Mails: 13780621366@163.com (Y.W.); luojian@hainu.edu.cn (J.L.); chguh@hainu.edu.cn (G.C.); 3 Qingdao Bluegranary Science & Technology Development Center Co., Ltd., Qingdao 266000, China; 4 State Key Laboratory of Biocontrol, Institute of Aquatic Economic Animals and Guangdong Provincial Key Laboratory for Aquatic Economic Animals, College of Life Sciences, Sun Yat-Sen (Zhongshan) University, Guangzhou 510275, China; E-Mails: lsszy@mail.sysu.edu.cn (Y.Z.); lsslxc@mail.sysu.edu.cn (X.L.); zsdxgl@163.com (L.G.)

**Keywords:** leptin genes, polymorphisms, genetic breeding, molecular markers, orange-spotted grouper (*Epinephelus coioides*), growth traits, association analysis

## Abstract

In mammals, leptin has been demonstrated to perform important roles in many physiological activities and to influence development, growth, metabolism and reproduction. However, in fish, its function is still unclear. Duplicate leptin genes, leptin-a and leptin-b, have been identified in the orange-spotted grouper. In the present study, the polymorphisms in the leptin-b gene of the orange-spotted grouper were detected, and the relation between these polymorphisms and 12 growth traits were analyzed. Six polymorphisms (including 3 single nucleotide polymorphisms (c.14G>A, c.93A>G, c.149G>A) in exon 1, 2 SNPs (c.181A>G, c.193G>A) in intron 1, and 1 SNP (c.360C>T) in exon 2) were identified and genotyped from 200 different individuals. The results revealed that the SNP c.149G>A was significantly associated with growth traits, that the heterozygous mutation genotype GA having negative effects on growth traits. However, the other five SNPs (c.14G>A, c.93A>G, c.181A>G, c.193G>A, c.360C>T) did not show significant associations with all the growth traits. Compared with our findings in leptin-a gene, the results suggested that the leptin-a hormone has more important physiological effects in fish bodies than the leptin-b type. Moreover, leptin genes were supposed to be one class of major candidate genes of regulating growth traits in the orange-spotted grouper.

## 1. Introduction

Leptin, the protein product of the *obese* (*ob* or *Lep*) gene, was a hormone synthesized by adipocytes that signals available energy reserves to the brain and acts on target tissues via receptor mediating mechanism [1,2]. In mammals, leptin has been demonstrated to play important roles in development, growth, metabolism and reproduction [3,4,5]. However, its function is still unclear in fish. Orthologs of mammalian Lep genes were isolated from several fish, such as pufferfish (*Takifugu rubripes*) [6], common carp (*Cyprinus carpio*) [7], zebrafish (*Danio rerio*) [8], Japanese medaka (*Oryzias latipes*) [9], grass carp (*Ctenopharyngodon idella*) [10], rainbow trout (*Oncorhynchus mykiss*) [11], Atlantic salmon (*Salmo salar*) [12], striped bass (*Morone saxatilis*) [13], goldfish (*Carassius auratus*) [14] and orange-spotted grouper [15]. In many teleost fish, the duplicate leptin genes have been detected, for instance zebrafish [8], Japanese medaka [9], and orange-spotted grouper [15]. 

In many farmed animals, the polymorphisms in leptin related genes have been demonstrated to be associated with the growth traits, particularly swine [16,17,18] and cattle [19,20,21,22,23,24]. The orange-spotted grouper (*Epinephelus coioides*) is a valuable culturing marine species, which has been farmed in many countries. Zhang (2013) identified duplicate leptin genes (leptin-a and leptin-b), in the orange-spotted grouper. The cDNAs of leptin-a and leptin-b gene were 671 bp and 684 bp in length, each containing 2 exons with 1 intron, and coding for proteins of 161 amino acids (AA) and 158 AA, respectively [15]. Recently, Wei *et al.* (2013) [25] reported that 6 SNPs in the leptin-a gene of the orange-spotted grouper were identified and confirmed three polymorphisms associated with growth traits, which is the first study about polymorphisms in fish leptin genes.

In the present study, polymorphisms of the leptin-b gene were identified and analyzed associations between the polymorphisms with growth traits. Then we compared the different findings between duplicate leptin genes in the orange-spotted grouper. This research is potentially useful for the application of leptin genes in marker-assisted selection (MAS) of the orange-spotted grouper and other cultured fish. 

## 2. Results and Discussion

### 2.1. Single Nucleotide Polymorphisms Identification and Genotyping

Six SNPs were determined by aligning the fragments of leptin-b gene from 200 individuals, consisting of three SNPs in exon 1 (c.14G>A, c.93A>G, c.149G>A), 2 SNPs in intron 1 (c.181A>G, c.193G>A) and 1 SNP exon 2 (c.360C>T). For the 4 SNPs in exons, c.14G>A was a missense mutation from arginine (CGG, Arg, R) to glutamine (CAG, Gln, Q); c.93A>G was a synonymous mutation of threonine (ACA→ACG, Thr, T); c.149G>A was a missense mutation from arginine (CGG, Arg, R) to glutamine (CAG, Gln, Q); c.360C>T was a synonymous mutation of leucine (CTC→CTT, Leu, L). The observed heterozygosity ranged from 0.060 to 0.525, and the expected heterozygosity ranged from 0.068 to 0.497, respectively. The chi-square tests revealed that all the loci were in the balance of Hardy-Weinberg (*p* > 0.05). Four SNPs (c.14G>A, c.93A>G, c.193G>A, c.360C>T) presented moderate polymorphisms, and the other two SNPs (c.149G>A, c.181A>G) presented low polymorphisms. In the experimental population, all heterozygous mutational genotypes were detected for the six SNPs, except recessive homozygosis mutational genotype for the SNP c.149G>A (Table 1).

### 2.2. Association Analysis with Growth Traits

The effects of leptin-b SNPs, and the analysis of association between different SNP genotypes and 12 growth traits were investigated (Table 2).

**Figure 1:**
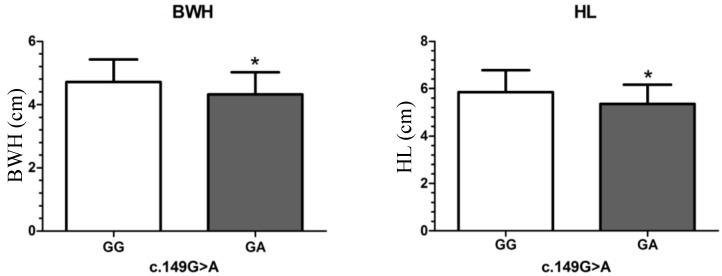
Significant differences in growth traits observed between different genotypes of c.149G>A. Data was shown as mean ± SD. Significant difference in growth traits of individuals with different genotypes were indicated with asterisks (* *p* < 0.05). BWH: body width, HL: head length.

For the missense mutation c.149G>A, the body width (BWH) and head length (HL) measurements of the GA genotype fish were significantly lower than those of the GG genotype fish (Figure 1A,B); the other 10 growth traits measurements of the GA genotype fish were also lower than those of the GG genotype fish, but the differences between them were not significant. These results revealed that the SNP c.149G>A were significantly effected the 12 growth traits, in which the heterozygous mutation of GA genotype having negative effects on growth traits. This SNP could be used for developing molecular marker in genetic breeding of orange-spotted grouper populations.

**Table 1:** Single nucleotide polymorphisms in the orange-spotted grouper leptin-b gene: genotype and allele frequencies, polymorphism information content, and chi-square tests of goodness-of-fit for Hardy-Weinberg equilibrium law in the experimental population.

SNP	Position	Mutation Type	Sample Size	Genotype Frequencies (%)			Allele Frequencies (%)		*H* o	*H* e	*p*-Value(χ^2^, HWE)	PIC ^a^
c.14G>A	Exon1	Missense	200	GG	GA	AA	G	A	0.525	0.497	0.731	0.374
	Locus14	R: CGG→Q: CAG	-	27.5	52.5	20.0	53.75	46.25	-	-	-	-
c.93A>G	Exon1	Synonymous	200	AA	AG	GG	A	G	0.430	0.428	0.997	0.336
	Locus93	T: ACA→ACG	-	47.5	43.0	9.5	69.00	31.00	-	-	-	-
c.149G>A	Exon1	Missense	200	GG	GA	AA	G	A	0.080	0.077	0.841	0.074
	Locus149	R: CGG→Q: CAG	-	92.0	8.0	0.0	96.00	4.00	-	-	-	-
c.181A>G	Intron1	Untranslated	200	AA	GA	GG	A	G	0.060	0.068	0.287	0.065
	Locus181	-	-	93.5	6.0	0.5	96.50	3.50	-	-	-	-
c.193G>A	Intron1	Untranslated	200	GG	GA	AA	G	A	0.400	0.416	0.863	0.329
	Locus193	-	-	50.5	40.0	9.5	70.50	29.50	-	-	-	-
c.360C>T	Exon2	Synonymous	200	CC	CT	TT	C	T	0.495	0.467	0.707	0.358
	Locus360	L: CTC→CTT	-	38.0	49.5	12.5	62.75	37.25	-	-	-	-

^a^ PIC = polymorphism information content; loci present high polymorphisms (PIC > 0.5); loci present moderate polymorphisms (0.25 < PIC < 0.5); loci present low polymorphisms (PIC < 0.25); HWE = Hardy-Weinberg equilibrium.

**Table 2:** Least squares means and standard deviations of growth traits between different genotypes of leptin-b SNPs in the orange-spotted grouper.

SNP	Genotypes	N		BWT (g)		BWH (cm)		OL (cm)		BL (cm)		TW (cm)		HL (cm)		CPW (cm)		CPL (cm)		SL (cm)		ED (cm)	ID (cm)	K (%)
c.14G>A	GG	55		87.89 ± 37.17		4.66 ± 0.72		17.78 ± 2.65		14.58 ± 2.22		2.72 ± 0.49		5.80 ± 0.88		1.58 ± 0.23		2.42 ± 0.49		1.16 ± 0.22		0.94 ± 0.08	0.92 ± 0.16	2.70 ± 0.33
	GA	105		92.86 ± 45.81		4.69 ± 0.72		18.07 ± 2.72		14.88 ± 2.38		2.76 ± 0.55		5.84 ± 0.92		1.62 ± 0.24		2.48 ± 0.50		1.20 ± 0.25		0.94 ± 0.09	0.93 ± 0.16	2.66 ± 0.31
	AA	40		92.60 ± 45.66		4.69 ± 0.77		17.97 ± 2.87		14.75 ± 2.56		2.75 ± 0.55		5.78 ± 1.01		1.59 ± 0.26		2.43 ± 0.54		1.19 ± 0.20		0.95 ± 0.12	0.96 ± 0.16	2.70 ± 0.32
	*p*-value	-		0.777		0.964		0.816		0.753		0.882		0.930		0.597		0.748		0.581		0.869	0.571	0.659
	GG/GA	-		1.000		1.000		1.000		1.000		1.000		1.000		1.000		1.000		0.895		1.000	1.000	1.000
	GA/AA	-		1.000		1.000		1.000		1.000		1.000		1.000		1.000		1.000		1.000		1.000	1.000	1.000
	AA/GG	-		1.000		1.000		1.000		1.000		1.000		1.000		1.000		1.000		1.000		1.000	0.889	1.000
c.93A>G	AA	95		93.74 ± 47.82		4.70 ± 0.77		18.09 ± 2.87		14.93 ± 2.52		2.78 ± 0.56		5.84 ± 0.97		1.61 ± 0.25		2.47 ± 0.53		1.19 ± 0.24		0.95 ± 0.10	0.95 ± 0.16	2.64 ± 0.34
	AG	86		88.11 ± 40.16		4.64 ± 0.72		17.74 ± 2.60		14.54 ± 2.24		2.69 ± 0.53		5.76 ± 0.92		1.60 ± 0.24		2.43 ± 0.49		1.18 ± 0.23		0.94 ± 0.09	0.92 ± 0.17	2.70 ± 0.30
	GG	19		94.95 ± 34.51		4.74 ± 0.51		18.37 ± 2.53		15.03 ± 2.19		2.86 ± 0.36		5.92 ± 0.75		1.62 ± 0.19		2.57 ± 0.44		1.20 ± 0.18		0.94 ± 0.07	0.96 ± 0.12	2.74 ± 0.30
	*p*-value	-		0.641		0.796		0.547		0.492		0.330		0.745		0.949		0.888		0.878		0.630	0.385	0.322
	AA/AG	-		1.000		1.000		1.000		0.834		0.748		1.000		1.000		1.000		1.000		1.000	0.645	0.760
	AG/GG	-		1.000		1.000		1.000		1.000		0.633		1.000		1.000		1.000		1.000		1.000	1.000	1.000
	GG/AA	-		1.000		1.000		1.000		1.000		1.000		1.000		1.000		1.000		1.000		1.000	1.000	0.620
c.149G>A	GG	184		93.02 ± 44.09		4.71 ± 0.72 ^a^		18.06 ± 2.73		14.86 ± 2.37		2.76 ± 0.53		5.86 ± 0.93 ^a^		1.61 ± 0.24		2.47 ± 0.50		1.19 ± 0.23		0.95 ± 0.09	0.94 ± 0.16	2.68 ± 0.32
	GA	16		73.25 ± 30.17		4.33 ± 0.70 ^b^		16.88 ± 2.42		13.78 ± 2.15		2.56 ± 0.51		5.36 ± 0.82 ^b^		1.50 ± 0.22		2.23 ± 0.50		1.14 ± 0.19		0.91 ± 0.09	0.89 ± 0.11	2.67 ± 0.30
	*p*-value	-		0.081		**0.041** *		0.095		0.081		0.147		**0.038** *		0.070		0.073		0.445		0.139	0.269	0.987
c.181A>G	AA	187		91.04 ± 43.05		4.68 ± 0.72		17.93 ± 2.65		14.75 ± 2.29		2.75 ± 0.53		5.80 ± 0.91		1.60 ± 0.24		2.45 ± 0.50		1.18 ± 0.23		0.94 ± 0.09	0.93 ± 0.16	2.68 ± 0.32
	AG	12		98.50 ± 51.91		4.72 ± 0.89		18.48 ± 3.92		15.18 ± 3.51		2.78 ± 0.69		6.09 ± 1.16		1.66 ± 0.26		2.60 ± 0.59		1.26 ± 0.25		0.96 ± 0.11	0.97 ± 0.21	2.63 ± 0.35
	GG	1		82.00 ± 0.00		4.60 ± 0.00		18.00 ± 0.00		14.50 ± 0.00		2.50 ± 0.00		6.00 ± 0.00		1.60 ± 0.00		2.30 ± 0.00		1.10 ± 0.00		0.90 ± 0.00	0.90 ± 0.00	2.69 ± 0.00
	*p*-value	-		0.828		0.979		0.802		0.829		0.875		0.557		0.741		0.570		0.502		0.790	0.783	0.870
	AA/AG	-		1.000		1.000		1.000		1.000		1.000		0.863		1.000		0.931		0.798		1.000	1.000	1.000
	AG/GG	-		1.000		1.000		1.000		1.000		1.000		1.000		1.000		1.000		1.000		1.000	1.000	1.000
c.193G>A	GG/AA	-		1.000		1.000		1.000		1.000		1.000		1.000		1.000		1.000		1.000		1.000	1.000	1.000
	GG	101		93.62 ± 47.51		4.71 ± 0.79		18.05 ± 2.89		14.88 ± 2.53		2.78 ± 0.58		5.83 ± 0.98		1.61 ± 0.25		2.47 ± 0.52		1.20 ± 0.24		0.95 ± 0.10	0.95 ± 0.17	2.66 ± 0.33
	GA	80		89.40 ± 40.78		4.67 ± 0.68		17.85 ± 2.64		14.66 ± 2.26		2.71 ± 0.48		5.81 ± 0.91		1.60 ± 0.24		2.46 ± 0.50		1.18 ± 0.23		0.94 ± 0.08	0.93 ± 0.17	2.68 ± 0.31
	AA	19		88.42 ± 30.70		4.59 ± 0.59		18.05 ± 2.23		14.68 ± 1.97		2.74 ± 0.47		5.80 ± 0.75		1.59 ± 0.21		2.34 ± 0.44		1.17 ± 0.17		0.93 ± 0.07	0.92 ± 0.11	2.72 ± 0.27
	*p*-value	-		0.771		0.804		0.883		0.823		0.695		0.986		0.916		0.584		0.842		0.494	0.667	0.736
	GG/GA	-		1.000		1.000		1.000		1.000		1.000		1.000		1.000		1.000		1.000		1.000	1.000	1.000
	GA/AA	-		1.000		1.000		1.000		1.000		1.000		1.000		1.000		1.000		1.000		1.000	1.000	1.000
	AA/GG	-		1.000		1.000		1.000		1.000		1.000		1.000		1.000		0.906		1.000		0.815	1.000	1.000
c.360C>T	CC	76		86.60 ± 37.50		4.61 ± 0.70		17.74 ± 2.76		14.54 ± 2.37		2.68 ± 0.48		5.78 ± 0.89		1.58 ± 0.22		2.41 ± 0.50		1.16 ± 0.21		0.94 ± 0.08	0.92 ± 0.16	2.68 ± 0.31
	CT	99		92.46 ± 46.06		4.70 ± 0.75		17.99 ± 2.64		14.81 ± 2.30		2.77 ± 0.57		5.80 ± 0.93		1.62 ± 0.26		2.46 ± 0.50		1.20 ± 0.25		0.95 ± 0.09	0.93 ± 0.16	2.67 ± 0.32
	TT	25		102.08 ± 48.72		4.82 ± 0.71		18.56 ± 2.94		15.34 ± 2.64		2.86 ± 0.52		6.01 ± 1.01		1.62 ± 0.26		2.58 ± 0.55		1.22 ± 0.20		0.96 ± 0.13	1.00 ± 0.17	2.68 ± 0.36
	*p*-value	-		0.288		0.410		0.428		0.340		0.312		0.537		0.552		0.326		0.428		0.522	0.119	0.944
	CC/CT	-		1.000		1.000		1.000		1.000		0.899		1.000		0.937		1.000		0.901		0.890	1.000	1.000
	CT/TT	-		0.969		1.000		1.000		0.966		1.000		0.938		1.000		0.795		1.000		0.785	0.261	1.000
	TT/CC	-		0.370		0.629		0.586		0.440		0.461		0.839		1.000		0.405		0.791		0.659	0.120	1.000

**BWT** = body weight, **BWH** = body width, **OL** = overall length, **BL** = body length, **TW** = trunk width, **HL** = head length, **CPW** = caudal peduncle width; **CPL** = caudal peduncle length, **SL** = snout length, **ED** = eyeball diameter, **ID** = interorbital distance, **K** = condition factor = 100 BWH/BL^3^; * Significant at the *p* < 0.05 level; ^a,b^ Multiple comparisons were performed using the least significant difference (LSD) test after Bonferroni correction adjustment; significant differences are shown using different superscripts (a *vs.* b) across the row; different superscripts within columns differ significantly (*p* < 0.05).

For the other 5 SNPs (c.14G>A, c.93A>G, c.181A>G, c.193G>A, c.360C>T), there were no significant differences for any growth traits between two genotypes. These results indicated that these 5 polymorphisms did not show significant associations with the 12 growth traits of the orange-spotted grouper. 

### 2.3. Discussion

Our results revealed that two missense mutations altering arginine to glutamine, c.14G>A and c.149G>A, existed in the leptin-b gene open reading frame of the orange-spotted grouper, with the SNP c.14G>A showing no significant associations and the SNP c.149G>A showing significant associations with growth traits in the orange-spotted grouper. According to the predicted amino acid sequence of the leptin-b gene [15], the amino acids altering by the SNP c.14G>A which existed in the signal peptide function fragment were detected. Signal peptide in *N*-domain, as the typical character of secreted protein, is the guide for transmembrane process and intracellular transport of secreted protein, while is cut and degradated by signal peptidase in endoplasmic reticulum [26]. Therefore, the amino acid altering by the SNP c.14G>A did not affect the functionality of leptin-b in fish bodies. We inferred that the amino acid altering by the SNP c.149G>A existed in an important function domain of leptin-b, and the amino acid variation interfered with the normal physiological effects of leptin-b in fish bodies, so that the SNP c.149G>A was associated with growth traits and the mutation genotype GA had negative effects.

In contrast with the results of leptin-a gene [25], we found that: (a) six SNPs were detected in the ORF areas of each duplicate leptin genes; (b) leptin-b gene presented higher polymorphism than leptin-a gene in the experimental population; (c) three mutation loci in leptin-a gene were associated with the growth traits, and one SNP in leptin-b gene showed significant associations with the growth traits. These findings indicated that the leptin-a gene presented more conservatism compared to the leptin-b gene, which suggested that the leptin-a hormone had more important physiological effects than the leptin-b type in the orange-spotted grouper. Some previous reports could support this conclusion. In the orange-spotted grouper, leptin-a mRNA was found to be expressed in the vast majority of fish tissues, while leptin-b mRNA hardly expressed in most of tissues except brain and ovary [15]. Furthermore, leptin-a mRNA was at generally higher levels than that of leptin-b in all the detected tissues [15]. As for the cloned leptin genes in fish, the leptin-a type was found to be present in the majority of species studied [27]. Based on the above, compared to the leptin-b gene, the leptin-a gene had more research value in genetic breeding, and leptin genes might be one class of major candidate genes of regulating growth traits in the orange-spotted grouper.

## 3. Experimental Section

### 3.1. Materials and Phenotypic Data Collection

The exhaustive descriptions of the experimental population were carried out as described by the reference [25]. The parent fish of the experimental population were selected from the first generation of a wild stock collected from the South China Sea. The experimental population was comprised by 200 juvenile fishes randomly selected from one net cage, which were maintained under identical living and environmental conditions from 10 December 2010 to 8 August 2011. Twelve phenotypic traits of every individual were measured, calculated and recorded, including BWT, BWH, OL, BL, TW, HL, CPW, CPL, SL, ED, ID and K. Total genomic DNA was extracted from fin clips according to the modified proteinase K/phenol extraction protocol [28]. The concentration of genomic DNA was determined by agarose gel electrophoresis using a UV spectrophotometer. 

### 3.2. PCR Amplification and SNP Identification

To detect leptin-b polymorphisms, he nucleotide coding sequences of the leptin-b gene ORF area from genomic DNA were determined. Based on *Epinephelus coioides* leptin-a mRNA nucleotide sequence (GenBank accession No. JX406148) by Primer Premier 5 software (PREMIER Biosoft, Palo Alto, CA, USA), two primers Leptin-b1F (TACAGGAGCACAGACACAGT) and Leptin-b1R (CCAGAGGAAGAGCATTATTG) were designed for amplification of the genomic DNA encoding leptin-b gene.

PCR amplifications were conducted in a final volume of 50 μL, containing 10 μL 5× PrimeSTAR GXL Buffer (Mg^2+^ Plus), 4 μL dNTPs (2.5 mM each), 1 μL of each primer (10 μM), 1 μL of PrimeSTAR GXL DNA Polymerase (1.25 U/μL, TaKaRa, Dalian, China) and 1 μL of genomic DNA (50–100 ng/μL). The PCR amplification reactions were performed with the following thermo cycle program: initial denaturation at 95 °C for 3 min followed 40 cycles of denaturation at 95 °C for 30 s, primer annealing at 55 °C for 1 min and primers extension at 72 °C for 1 min. At the end of the last cycle, primers extension was conducted at 72 °C for 10 min. Amplification results were verified by 1.5% agarose gel electrophoresis. PCR fragments of the predicted size were cut and purified from gels with an agarose gel DNA Extraction kit (D823A, TaKaRa). 

### 3.3. SNP Identification and Genotyping

The sequence of the amplified DNA fragment was determined with an ABI3100 DNA sequencer by the Shenzhen Genomic Institute (Shenzhen, China). Sequence mutations between different individuals were detected using SeqMan Pro version 7.1.0 (44.1) (DNASTAR Inc., Madison, WI, USA). SNPs were identified and genotyped by analyzing and comparing chromatogram files using Chromas version 2.33 (Technelysium Pty Ltd., South Brisbane, Australia).

### 3.4. Statistical Analysis

Allele and genotype frequencies were calculated using a simple allele counting method. Hardy-Weinberg equilibrium was tested for goodness-of-fit by comparing expected and observed genotype frequencies using a chi-square test. 

Association analyses between genotypes of leptin-b gene and 12 growth traits were performed using general linear model (GLM) procedure with SPSS 20.0 software (IBM, Armonk, NY, USA). The following statistical model: *Y* = *u* + *G* + *e* was performed, where *Y* is the phenotypic value of each trait; *u* is population mean value of each growth traits, *G* is the fixed genotype effect of each SNP, and *e* is the random error effect. Multiple comparisons in growth traits between different genotypes were tested using the least significant difference (LSD) method with Bonferroni correction adjustment.

## 4. Conclusions

Six SNPs in the leptin-b gene ORF area of the orange-spotted grouper were identified. Association analyses with 12 growth traits revealed that: the SNP c.149G>A was significantly associated with growth traits, with the heterozygous mutation genotype GA having negative effects on growth traits; the other 5 SNPs (c.14G>A, c.93A>G, c.181A>G, c.360C>T) didn’t show significant associations with all of the growth traits. Our findings in duplicate leptin genes of the orange-spotted grouper suggested that the leptin-a hormone had more important physiological effects than the leptin-b type in fish bodies, and leptin genes might be one class of major candidate genes of regulating growth traits in the orange-spotted grouper.
